# Integrating mercury research and policy in a changing world

**DOI:** 10.1007/s13280-017-1010-y

**Published:** 2018-01-31

**Authors:** Celia Y. Chen, Charles T. Driscoll

**Affiliations:** 1 Department of Biological Sciences, Dartmouth College, Hanover, NH USA; 2 Department of Civil and Environmental Engineering, Syracuse University, Syracuse, NY 13244 USA

Mercury (Hg) is a complex, multifaceted contaminant. Methylmercury (MeHg), the more bioavailable and toxic form, biomagnifies and drives most human health advisories and concerns for wildlife impacts. Moreover, Hg transport, transformations, bioaccumulation, and exposure are affected by numerous interacting processes and phenomena (e.g., climate change, nutrient loading, land use/cover, food web dynamics, human behavior and decisions). Approximately, two-thirds of the Hg entering the environment comes from anthropogenic sources including mining, industrial activities, coal combustion, and incinerators, with the remaining supplied from natural sources (Lindberg et al. 2007; UNEP 2013; Driscoll et al. 2013; Obrist et al. 2018). Mobilized Hg readily interacts with the biosphere and eventually is transported to all water bodies. Hg is ubiquitous and reaches levels of concern in fish not only in contaminated environments, but also in remote and otherwise pristine ecosystems (Driscoll et al. 2007, 2013; Chen et al. 2012a, b). Fluxes of Hg in aquatic ecosystems have increased substantially with industrialization (Mason et al. 1994, 2012; Driscoll et al. 2013; Obrist et al. 2018), and Hg now occurs in fish and shellfish throughout the biosphere at levels that can pose risks to humans and wildlife (Fitzgerald and Clarkson 1991; Grandjean et al. 2005; Driscoll et al. 2007; Mergler et al. 2007; Chen et al. 2008a; Karagas et al. 2012; Eagles-Smith et al. 2018).

MeHg that affects human and wildlife health can originate from sources both nearby and far away. Mercury is predominantly transported through the atmosphere in its elemental form, which has an atmospheric lifetime of approximately 6 months to a year which means it is truly a global pollutant (Driscoll et al. 2007, 2013; Giang and Selin 2016; Obrist et al. 2018). Mercury released in more reactive oxidized forms can enter ecosystems closer to sources (Selin et al. 2008; Selin 2009; Hsu-Kim et al. 2018). Mercury can readily cycle among the atmosphere, land, and ocean, and as a result, past and present emissions continue to contaminate on timescales of decades to centuries (Amos et al. 2013). Mercury currently depositing to ecosystems represents a combination of (1) current anthropogenic emissions (e.g., from coal-fired power generation, used in artisanal and small-scale gold mining (ASGM)), (2) natural sources, and (3) legacy contamination from historical anthropogenic emissions. This global biogeochemical processing of Hg, involving the emission and cycling of different Hg forms is also influenced by meteorological and human perturbations. This complexity challenges our ability to detect changes in Hg in the environment and determine the factors responsible for these changes. A critical need is to establish and maintain monitoring programs to evaluate the effectiveness of Hg control measures. Scientific research is essential to better understand and quantify the sources and environmental processing of Hg in ways that inform policy efforts to manage its risks (Selin 2014).

MeHg is the most toxic form of Hg, and the main exposure route for MeHg to humans and wildlife is through consumption of fish and shellfish (Rice et al. 2000; Karagas et al. 2012; Oken et al. 2012; Eagles-Smith et al. 2018). Toxicological effects of MeHg are of special public concern to high-risk populations including women and young children. Unborn fetuses are at greatest risk for neurological and developmental impairment since Hg can pass through the placenta to the fetal brain. People who consume elevated amounts of fish or shellfish contaminated with MeHg are expected to have a higher body burden of the metal when compared to others (Mahaffey and Mergler 1998; Karimi et al. 2012, 2014, 2016). The developing human nervous system is a sensitive target organ system for low-dose MeHg exposure (Schober et al. 2003; Oken et al. 2008). The global health risks to humans and wildlife that result from exposure to this neurotoxin are significant, especially in populations that depend on fish for subsistence. Human exposure to Hg at levels with potential risks has been found in fish-eating populations all over the world and across all socioeconomic spectra (Mahaffey et al. 2004; Grandjean et al. 2005; Costa et al. 2012; Karagas et al. 2012; Kirk et al. 2012). However, regulating dietary exposure by reducing fish consumption poses a particular challenge (Oken et al. 2003, 2012; Groth 2010). Fish are highly nutritious with significant benefits to human health, a culturally important food source for many populations, and a significant component of the global economy (Pirrone and Mahaffey 2005; Gribble et al. 2016). Thus, the science of Hg cycling and bioaccumulation in fish, and its management in the environment are a focus of attention worldwide.

The control and management of Hg pollution require both global and local efforts. The Minamata Convention, a global treaty on Hg, was ratified in August 2017 and is now entering the implementation phase. At the same time, local efforts worldwide are in progress to remediate Hg contaminated sites. The Convention requires that countries around the world control both new and existing sources and monitor the effectiveness of those controls. In the US, the Mercury and Air Toxics Rule is being implemented which will limit primary anthropogenic emissions. In many countries, the use of Hg in ASGM is under investigation as the magnitude of associated Hg releases and effects has been underestimated (UNEP 2013). At the same time, uncertainty remains in the levels of exposure that result in adverse effects of Hg on wildlife and human health. While these initiatives are important steps to mitigate Hg contamination, the extent and rate of potential recovery is unclear because of uncertainties in our understanding of Hg transport, cycling and trophic transfer in the face of global change.

In July of 2017, the 13th International Conference on Mercury as Global Pollutant (ICMGP) was held in Providence Rhode Island, USA. The theme of the conference was “understanding the multiple factors that accelerate and attenuate recovery of mercury contamination in response to environmental inputs on local to global scales.” The conference brought together over 1000 delegates from over 50 countries and included participants from industry, government, research institutions, NGOs and academia. The technical program of the conference reflected the latest advances, highlighted critical understanding and provided opportunity for active discussion of the science of Hg and innovative strategies for its management.

Previous Hg conferences organized synthesis activities taking various forms. For example, the Madison (2006) conference facilitated a group of synthesis papers and The Madison Declaration on Mercury Pollution (2007), and the Halifax conference (2011) drew from plenary talks in a single synthesis paper (Driscoll et al. 2013). In addition, past Hg synthesis efforts have been led by the Hubbard Brook Research Foundation (Driscoll et al. 2007) and the Dartmouth Toxic Metals Superfund Research Program (Chen et al. 2008; Chen and Wilcox 2008; Chen 2012; Chen et al. 2012a, b). For this ICMGP 2017 synthesis, four plenary synthesis themes were identified and papers developed to connect Hg science to regulatory issues and policy implementation, as well as communication of science to stakeholders.

In summer of 2016, lead authors for the ICMGP 2017 synthesis were invited to assemble author teams to develop and write the papers in 2016–2017. The draft papers were then made available for conference participants in advance of the meeting. A synthesis workshop sponsored by the National Institute of Environmental Health Sciences and the Dartmouth Toxic Metals Superfund Research Program was held prior to the ICMGP for face-to-face interaction between the synthesis author groups and Hg policy and management stakeholder groups. These papers were featured during the plenary sessions of the ICMGP 2017 and were also summarized in 2-page summary fact sheets (http://mercury2017.com/program/synthesis-effort/) for distribution to delegates at the first Conference of Parties of the Minamata Convention, held September 24–29 in Geneva, Switzerland. Following the ICMGP 2017 and prior to submission, the authors of the papers revised their drafts in response to discussion and comments from conference participants.

The four plenary themes that structured the ICMGP 2017 were organized around a series of questions: (1) How is global Hg cycling changing in response to perturbations (e.g., climate change, emissions control)? (2) How is Hg cycling (and bioaccumulation) changing in specific places in response to perturbations (e.g., climate change, remediation, nutrient control, urbanization)? (3) What is the relative risk of Hg exposure to human health and wildlife in the context of other risks/stressors? and (4) How can scientific knowledge contribute to the implementation and effectiveness evaluation of the Minamata Convention? Papers 1–3 review and synthesize the most current Hg research and address the role of environmental perturbations and stressors that interact to reduce or enhance the fate and effects of Hg in the environment. Synthesis paper 4 addresses the role of science in informing the implementation of the Minamata Convention. The findings of each of the papers are summarized below and are published in this special section of *Ambio*.

Synthesis 1 “A review of global environmental mercury processes in response to human and natural perturbations: Changes of emissions, climate and land use” (Obrist et al. 2018) summarizes current understanding of the global cycling of Hg between major global reservoirs, i.e., atmosphere, terrestrial environments, and aquatic ecosystems. The most recent estimates of Hg concentrations and pool sizes in these compartments are provided and exchange processes within and between these reservoirs are described. While atmospheric concentrations and wet deposition of Hg have declined in the North America and Europe, no declines have been measured in the southern hemisphere and atmospheric Hg loads are increasing in East Asia. As a result, ocean concentrations have declined in the North Atlantic, while they are increasing in the Pacific. Meanwhile, half of the total wet deposition of Hg is predicted to be occurring in the tropical oceans although these predictions are poorly constrained by measurements. The major source of Hg to the oceans is atmospheric deposition, while ocean evasion is a large source of Hg^0^ to the atmosphere. Terrestrial environments are net sinks of atmospheric Hg and half of aquatic releases from land are estimated to occur in China and India draining into the West Pacific and North Indian Oceans. This synthesis paper also discusses projections of large impacts of global change on Hg cycling due to remobilization of legacy pollution in soils and oceans.

Synthesis 2, “Challenges and opportunities for managing aquatic mercury pollution in altered landscapes” (Hsu-Kim et al. 2018), provides a synthesis of the scientific understanding of how Hg cycling in the aquatic environment is influenced by natural and anthropogenic perturbations at the local scale. These perturbations include watershed loadings, deforestation, reservoir and wetland creation, rice production, urbanization, mining and industrial point source pollution, and remediation of contaminated sites. Policies and management opportunities are discussed that could lessen both MeHg levels in biota and exposure to humans, such as technologies for remediation and social and political issues associated with ASGM. The authors call for meaningful application of Hg science for stakeholders including those communities living near Hg-polluted sites, decision makers of environmental policies, and scientists and engineers developing watershed management solutions related to Hg pollution.

Synthesis 3, “Modulators of mercury risk to wildlife and humans in the context of rapid global change” (Eagles-Smith et al. 2018), provides understanding of ecological and human health risks from Hg and how health risks are impacted by complex environmental cycling, variable toxicokinetics, and the diverse effects of this element. The authors identify three domains of drivers that influence Hg risk to humans and other organisms. In Domain 1, extrinsic globally occurring drivers such as land use changes, hydrologic management, invasive species, and climate change interact with mechanisms (habitat use, bioenergetics, primary production and food web structure) to alter MeHg movement in food webs. In Domain 2, external drivers (e.g., socioeconomic factors of fish consumption, ASGM) alter MeHg exposures and intrinsic drivers (genetics and gastrointestinal assimilation) that affect human exposure. In Domain 3, extrinsic and intrinsic drivers including diet, nutrition, co-exposures to other contaminants or diseases, genetics, and microbiome modulate adverse outcomes of Hg. The future implementation of Hg pollution control policies such as the Minamata Convention requires an understanding of these drivers and mechanisms in order to properly evaluate the effectiveness of Hg reduction activities on ecological and human health risk.

The 4th synthesis on the Minamata Convention, “Linking science and policy to support the implementation of the Minamata Convention on Mercury” (Selin et al. 2018), identifies and examines areas in which the scientific community can apply knowledge in support of Hg abatement and realize the objective of the Minamata Convention. The paper offers specific guidance for research scientists to connect with international, national, and local efforts in three focal areas: (1) uses, emission, and releases of Hg; (2) support, awareness raising, and education; and (3) impacts and effectiveness evaluation. The authors suggest that developing and newly industrialized countries will likely be where the most wide-ranging policies will be needed since they currently dominate global Hg uses and releases. Building implementation capacity in these countries and supporting technology transfer will be critical to the success of the Convention. The authors encourage the Hg science community to actively engage with the policy implementation of the Minamata Convention.

Scientific research on Hg transport and fate in the environment and risk to human and wildlife health is growing at a time when international and national policy approaches are needed and being formulated (Evers et al. 2016; Sunderland et al. 2016). The time is optimal for translating the science of Hg pollution and providing guidance to policymakers so that decisions made under the Minamata Convention and national and local efforts to control and mitigate Hg releases and limit exposure can be based on the most rigorous and current scientific research. These four synthesis papers represent collective findings of the international Hg science and policy communities. They will provide a basis in the coming years for developing policy to minimize environmental effects and exposures of Hg.
